# Fast Absorbent and Highly Bioorthogonal Hydrogels Developed by IEDDA Click Reaction for Drug Delivery Application

**DOI:** 10.3390/ma15207128

**Published:** 2022-10-13

**Authors:** Soo-Bin Joo, Muhammad Gulfam, Sung-Han Jo, Yi-Jun Jo, Trung Thang Vu, Sang-Hyug Park, Yeong-Soon Gal, Kwon Taek Lim

**Affiliations:** 1 Department of Smart Green Technology Engineering, Pukyong National University, Busan 48513, Korea; 2 Department of Industry 4.0 Convergence Bionics Engineering, Pukyong National University, Busan 48513, Korea; 3 Department of Fire Safety, Kyungil University, Gyeongsan 38428, Korea

**Keywords:** hyaluronic acid, injectable, porous hydrogels, curcumin, IEDDA click-reaction, drug delivery

## Abstract

In this work, we engineered highly biocompatible and fast absorbent injectable hydrogels derived from norbornene (Nb)-functionalized hyaluronic acid (HA-Nb) and a water-soluble cross-linker possessing tetrazine (Tz) functional groups on both ends of polyethylene glycol (PEG-DTz). The by-product (nitrogen gas) of the inverse electron demand Diels–Alder (IEDDA) cross-linking reaction carved porosity in the resulting hydrogels. By varying the molar ratio of HA-Nb and PEG-DTz (Nb:Tz = 10:10, 10:5, 10:2.5), we were able to formulate hydrogels with tunable porosity, gelation time, mechanical strength, and swelling ratios. The hydrogels formed quickly (gelation time < 100 s), offering a possibility to use them as an injectable drug delivery system. The experimental data showed rapid swelling and a high swelling ratio thanks to the existence of PEG chains and highly porous architectures of the hydrogels. The hydrogels were able to encapsulate a high amount of curcumin (~99%) and released the encapsulated curcumin in a temporal pattern. The PEG-DTz cross-linker, HA-Nb, and the resulting hydrogels showed no cytotoxicity in HEK-293 cells. These fast absorbent hydrogels with excellent biocompatibility fabricated from HA-Nb and the IEDDA click-able cross-linker could be promising drug carriers for injectable drug delivery applications.

## 1. Introduction

In the last few decades, numerous drug delivery systems such as polymersomes, liposomes, nanoparticles, micelles, and hydrogels have been formulated for the delivery or controlled release of cytotoxic or low bioavailable therapeutic agents [1]. Specifically, hydrogels, which are cross-linked and three-dimensional network configurations of hydrophilic polymer chains and can engross a huge volume of water/biological fluids or can encapsulate therapeutic molecules in their internal networks, have received great attention in drug delivery. Regardless of their benefits, traditional hydrogels require a surgical procedure for their implantation to a malignant site. To address this problem, injectable hydrogels which can be employed by nominally invasive means and can be molded into any shape have received great interest in drug delivery. These injectable hydrogels not only mimic extremely hydrated 3D microenvironment of native extracellular matrix, but can also deliver therapeutic molecules to diseased sites. The injectable hydrogels could be engineered by synthetic or natural polymers using sol-gel phase transition [2], in-situ polymerization [3], and physical or chemical cross-linking strategies [4].

From a bio-imitation perspective, an ideal injectable hydrogel should be fast absorbent, i.e., can absorb a large amount of water and should possess interlocked pores to facilitate proficient supply of oxygen, nutrients, growth factor/therapeutic compounds and/or waste diffusion, mimicking the native tissue-environment [5]. Previous reports have shown that highly porous hydrogels have several benefits over their non-porous counterparts [6]. Natural polymer-based porous hydrogels could be developed by using various additives and approaches. For instance, porous scaffolds derived from gelatin were developed by an emulsion template method [7]. For this purpose, gelatin droplets were initially engineered by emulsification, and subsequently stabilized by genipin cross-linkers, generating microspheres, which were later employed as a template for the development of gelatin-based porous hydrogels. It was demonstrated that hydrogels possessed macro-porous and interlocked networks and that the pore-sizes could be programmed by changing the diameters of the microspheres. However, the genipin cross-linker could comprise the biocompatibility of the resulting porous materials. Porogen leaching is another method to carve porosity in hydrogels, which implicates mixing of inorganic salts (e.g., sodium chloride, sugar crystals, etc.) with a polymer solution prior to casting into a mold [8]. The hydrogels are then lyophilized and the salt particles are rinsed out to generate porosity in the hydrogels. Nevertheless, large difference in pore sizes, absence of interconnectivity, and uneven pore geometry have restricted the use of this technique. Lyophilization has also been widely exploited to form porous hydrogels [9]. Nevertheless, poor control over pore-sizes and low structure stability of the resulting porous hydrogels renders the lyophilization technique less practical.

Recently, porous hydrogels prepared through an in-situ bubble generating procedure were developed from hyaluronic acid (HA) and cystamine via carbodiimide chemistry [10]. Specifically, 1-ethyl-3-(3-dimethylaminopropyl)-carbodiimide (EDC) and N-hydroxy-succinimide (NHS) released carbon dioxide gas as a by-product during the coupling reaction, generating pores in the hydrogel networks. This approach is facile and simple, the EDC and NHS coupling agents, however, might induce unwanted cytotoxicities, and are not considered safe for injectable drug delivery systems. It is noteworthy that most of the above-mentioned approaches either employ cytotoxic reagents or use destructive techniques which are not suitable for injectable hydrogels, and omits any chance of injecting them into the diseased site. Therefore, porous and injectable hydrogels possessing better biocompatibility are highly needed for biomedical applications.

Various polymeric materials can be used to make porous and injectable hydrogels. Hyaluronic acid (HA), which is a natural linear polysaccharide composed of alternating units of the repeating disaccharide, β-1,4-D-glucuronic acid and β-1,3-N-acetyl-D-glucosamine, have received much attention in drug delivery due to its exceptional cytocompatibility and biodegradability [11,12,13,14]. In spite of a number of advantages, HA has feeble mechanical properties and a quick degradation profile [15]. The mechanical strength and degradation profiles of HA have been improved by covalent cross-linkers, such as glutaraldehyde, genipin, and EDC, previously. Although these cross-linkers have produced stable hydrogels, their high cytotoxicities have been reported [16].

Recently, click chemistry has been considered as a promising approach to engineer covalently cross-linked hydrogels [17,18]. In particular, the inverse electron demand Diels-Alder (IEDDA) reaction between norbornene (Nb) functional groups and tetrazine (Tz) functional groups has been regarded as a highly biocompatible approach for the synthesis of hydrogels [19]. The IEDDA reaction possesses high chemo-selectivity and ultrafast kinetics [20], and can be performed in catalyst-free environment at mild conditions. Interestingly, the sole by-product of this reaction is nitrogen gas, which is non-toxic, making it suitable for injectable drug delivery applications [21,22,23]. In addition, the liberated nitrogen gas can generate pores in the hydrogels [20], and this porous structure can modulate the drug encapsulation and release performance of the resulting hydrogels [24]. Furthermore, depending upon the amount of Tz and/or cross-linkers, the gelation times of hydrogels could be adjusted to few seconds to several minutes, making these materials injectable.

**Scheme 1:**
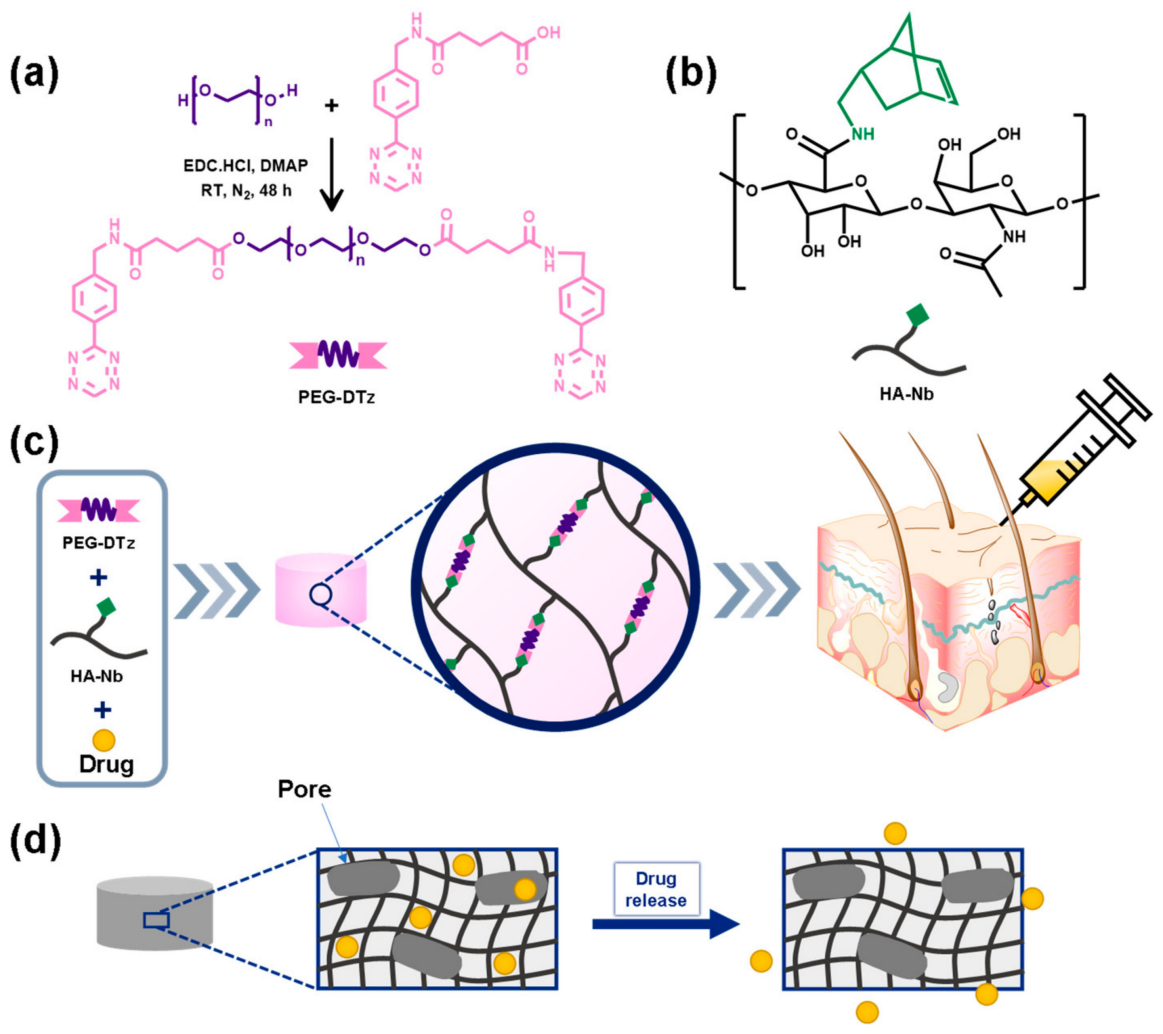
Graphical representation of (**a**) Synthesis of PEG-DTz, (**b**) Chemical structure of HA-Nb, (**c**) Schematic diagram representing development of injectable hydrogels, and (**d**) Mechanism of curcumin release from the porous hydrogel.

In this study, highly biocompatible and fast absorbent injectable hydrogels were developed by using Nb-functionalized HA (HA-Nb) and a water-soluble cross-linker (polyethylene glycol-ditetrazine, PEG-DTz) possessing Tz functional groups on both ends of PEG. The PEG-Tz was synthesized by a simple carbodiimide reaction (Scheme 1a). Since PEG-DTz and HA-Nb (Scheme 1b) are highly water-soluble precursors and the IEDDA click reaction is highly bioorthogonal, it was possible to produce hydrogels with excellent biocompatibility. Three types of hydrogels were developed by changing the mol. ratios of the cross-linker (Nb:Tz = 10:10, 10:5, 10:2.5). The hydrogels formed fast (sol-gel time < 100 s), offering a possibility to use them as injectable drug delivery systems (as illustrated in Scheme 1c). Nitrogen gas liberated during the IEDDA reaction resulted in a highly porous structure. The porous structure could regulate the drug release from the hydrogels as illustrated in Scheme 1d. Experimental data of the prepared hydrogels showed rapid swelling owing to the existence of PEG chains and highly porous architectures of the hydrogels. Curcumin (a hydrophobic bioactive drug derived from turmeric) was used as a model therapeutic molecule to assess the drug loading and release behavior of the hydrogels. The hydrogels were able to encapsulate a high amount of curcumin (~99%) and released it in a temporal pattern. The PEG-DTz cross-linker, HA-Nb and the resulting hydrogels showed no cytotoxicity in HEK-293 cells, exhibiting excellent biocompatibility of these hydrogels. These fast absorbent and injectable hydrogels with excellent biocompatibility could be promising drug carriers for injectable drug delivery applications.

## 2. Materials and Methods

### 2.1. Materials

Polyethylene glycol (OH-PEG-OH, Mn = 1000), 4-(dimethylamino)pyridine (DMAP, 99%), sodium chloride (NaCl, 99%), NHS (98%), triethylamine (TEA, 99%) were purchased from Sigma Aldrich (Waltham, M, USA). EDC.HCl (99%) and 5-Norbornene-2-methylamine (Nb-NH2, 98%), were acquired from Tokyo Chemical Industry (TCI, Tokyo, Japan). Acetone, dichloromethane (DCM), diethyl ether, and ethyl acetate, were obtained from Duksan Pure Chemicals (Siheung, Korea). Sodium hyaluronate (HA, Mw ~800 kDa) was purchased from Bioland Korea (Chungbuk, Korea). 4- (Aminomethyl)benzonitrile hydrochloride (98%) was purchased from Matrix Scientific. Curcumin (95%) was purchased from Alfa Aesar (Haverhill, MA, USA).

### 2.2. Measurements

^1^H NMR analyses were carried out using a JEOL NMR spectrometer (JNM ECZ-400, JEOL, Akishima-shi, Japan). Fourier transform infrared (FTIR) spectra were obtained using an Agilent Cary640-FTIR. The surface and cross-sectional structure of hydrogels was observed by using a low vacuum scanning electron microscope (SEM, JEOL, Gatan, JSM-6490LV, Pleasanton, CA, USA). The UV-vis spectra were recorded on an Optizen POP UV-vis spectrophotometer. The rheology of hydrogels was investigated using a Discovery HR-2 hybrid rheometer (TA instrument) mounted on a flat steel plate.

### 2.3. Methods

#### 2.3.1. Synthesis and Characterization of Precursors

##### Synthesis of (4-(Cyano) Benzylamino)-5-Oxopentanoic Acid

The (4-(cyano) benzylamino)-5-oxopentanoic acid was obtained following a previously published protocol after minor changes [25]. Briefly, 4- (aminomethyl) benzonitrile hydrochloride (2 g, 11.86 mmol) was mixed with 100 mL of extra pure acetonitrile in a two-neck round bottom flask (RBF). After purging with nitrogen for 30 min, TEA (5 mL, 35.58 mmol) was introduced int the RBF. After stirring for another 30 min, glutaric anhydride (1.48 g, 13.04 mmol) was dispersed in acetonitrile and then was incorporated with the mixture. The reaction was refluxed at 85 °C for 24 h. After evaporating the solvent by a rotary evaporator, solid crystals were suspended in 100 mL of DI water. Next, 1N HCl was introduced to the mixture to adjust the pH to 3 and then it was extracted 3 times with 100 mL of ethyl acetate. The fractions of the ethyl acetate were combined and washed again with water (100 mL) and brine (100 mL). The mixture was treated with anhydrous MgSO_4_ to remove the water residues and was filtered. Finally, the ethyl acetate was evaporated using a rotary evaporator to obtain a white solid (91% yield). The structure of (4-(cyano) benzylamino)-5-oxopentanoic acid was confirmed by ^1^H NMR (Supplementary Materials Figure S1a) and ^13^C NMR (Supplementary Materials Figure S1b). (^1^H NMR, 400 MHz, DMSO-d_6_, δ): 12.05 (s, 1H), 8.44 (t, J = 5.9 Hz, 1H), 7.78 (d, J = 8.5 Hz, 2H), 7.42 (d, J = 8.6 Hz, 2H), 4.33 (d, J = 6.0 Hz, 2H), 2.26–2.15 (m, 4H), 1.79–1.69 (m, 2H).

##### Synthesis of Tetrazine(benzylamino)-5-Oxopentanoic Acid (Tetrazine-COOH)

The Tetrazine-COOH) was obtained by following a previously published protocol after minor changes [25]. Briefly, (4-cyanobenzylamino)-5-oxopentanoic acid (1 g, 4.08 mmol) and formamidine acetate salt (2.12 g, 20.39 mmol) were mixed in RBF. After adding zinc triflate (0.074 g, 0.20 mmol) into the mixture, anhydrous hydrazine (3.26 mL, 102 mmol) was incorporated dropwise. After vigorous gas release, the resulting viscous slurry was stirred overnight at room temperature (RT). Next, sodium nitrite (2.82 g, 40.80 mmol) was dissolved in 5 mL of DI H_2_O and was incorporated with the reaction solution. Next, the RBF was moved to an ice bath (0 °C) and 1N HCl was carefully introduced into the solution dropwise (at this stage vigorous gas release was noted). When around 150 mL of 1N HCl was consumed and the pH approached to 3, the solution turned into bright pink and the generation of gas stopped. Subsequently, the suspension was extracted with 100 mL DCM (3 times) and the organic phase was washed with brine (3 × 100 mL). After drying with anhydrous MgSO_4_ and filtering, DCM was evaporated by using a rotary evaporator. Finally, the solid was purified by recrystallization in cold isopropyl alcohol to obtain Tetrazine-COOH as a bright pink solid (53% yield). The structure of Tetrazine-COOH was confirmed by ^1^H NMR (Supplementary Materials Figure S2a) and ^13^C NMR (Supplementary Materials Figure S2b). (^1^H NMR, 400 MHz, DMSO-d_6_, δ): 12.06 (s, 1H), 10.58 (s, 1H), 8.52–8.40 (m, 3H), 7.53 (d, J = 8.6 Hz, 2H), 4.40 (d, J = 5.9 Hz, 2H), 2.28–2.17 (m, 4H), 1.77 (p, J = 7.3 Hz, 2H).

##### Synthesis of PEG-DTz

Typically, tetrazine-COOH (198.86 mg, 0.66 mmol) was mixed with a solvent mixture containing 2 mL of DMSO and 10 mL of DCM in a RBF, and then the resulting mixture was degassed under stirred at 0 °C. Next, DMAP (91.62 mg, 0.75 mmol) was dissolved in 10 mL of DCM and was introduced while maintaining an inert environment. Next, EDC.HCl was mixed with 10 mL of DCM and then was introduced dropwise into the reaction flask. The solution was stirred at RT for 1 h to activate carboxylate functionality. Afterwards, PEG (300 mg, 0.3 mmol) was mixed with 2 mL of DCM and then was incorporated with reaction mixture. The contents were then stirred under nitrogen at RT for 48 h and then mixed with 70 mL of DCM. The diluted mixture was washed 3 times with DI H_2_O (125 mL) and once with brine (100 mL). The DCM phase was dried with anhydrous MgSO_4_ and filtered with a filter paper, and then DCM was evaporated by using a rotary evaporator. Finally, the product was precipitated in 40 mL of diethyl ether. The resulting crystals were filtered and dried in under low pressure for 1 d to acquire PEG-DTz as a pink solid. (67.13% yield), ^1^H NMR (400 MHz, DMSO-D6) δ = 10.58 (s, 2 H), 8.52–8.40 (m, 6 H), 7.53 (d, 4 H), 4.40 (d, 4H), 4.13 (t, 4 H), 3.68 (t, 4 H), 3.51 (s, 91 H), 2.34 (t, 4 H), 2.23 (t, 4 H), 1.80 (t, 4 H).

##### Synthesis of HA-Nb

HA-Nb was synthesized following a previous protocol [21]. Briefly, HA (2000 mg, 0.0025 mmol) was mixed with 200 mL of DI water until a homogenous solution was obtained. Next, EDC.HCl (475.40 mg, 2.48 mmol) and NHS (427.84 mg, 3.72 mmol) were incorporated with the suspension and stirred for 1 h. After adding Nb-NH2 (458 mg, 3.72 mmol, dissolved in 5 mL of DMSO) to the solution, the mixture was stirred for 24 h at RT. Subsequent, NaCl (1.5 g) was mixed with 5 mL of DI water and incorporated with the mixture. After stirring for 10 min, the solution was precipitated in an excess of acetone. After filtering, the sponge-like solid product was dried under reduced pressure. Next, the solid was re-dispersed in 200 mL of DI water and shifted to a dialysis bag (MWCO = 14 kDa) and dialyzed in DI water for 2 d. After freezing the mixture, it was lyophilized to acquire the dry HA-Nb with 18.55% degree of substitution (measured by ^1^H NMR). The structure of the HA-Nb was further characterized by FTIR analysis.

#### 2.3.2. Preparation of Hydrogels

The cross-linked hydrogels based on the IEDDA click reaction were developed by a one-step mixing of the PEG-DTz cross-linker and HA-Nb. Hydrogels with 3 cross-linking conformations (as presented in Table 1) were prepared to acquire the hydrogels with varying physicochemical properties. Briefly, 200 μL of a 2% HA-Nb solution (prepared by dissolving HA-Nb in DI water) and 35 μL of a PEG-DTz solution (in DI water) having various concentration were gently mixed in an Eppendorf for 15 s by means of a vortex. The solution mixture was instantly transferred to a syringe (25G needle) to test the injectability.

#### 2.3.3. Rheological Properties of Hydrogels

The viscoelastic behaviors of hydrogels were assessed by a rheometer. Specifically, sol-gel transformation times (gelation times) were calculated by measuring the storage modulus G“ and loss modulus (G”) of hydrogels as function of step-time. The sol-gel transformation time could be considered when G’ and G” intersect each other. Similarly, an angle frequency sweep test and amplitude sweep test were carried out to assess the mechanical strength and shear thinning properties of hydrogels.

#### 2.3.4. Swelling Studies

To measure swelling ratios, hydrogels were lyophilized and weighed before immersing them in 3 mL of PBS (pH = 7.4). After pre-determined times, hydrogels were taken out from the swelling medium and were carefully treated with a tissue paper to eliminate extra water presented on the upper surface of hydrogels before measuring their weights. The non-lyophilized hydrogels were also assessed for their swelling behavior. The swelling ratios of hydrogels were measured by using the following equation:

(1) $$\mathrm{Swelling}\;\mathrm{Ratio}\;\left(\%\right)=\frac{M_{s}-M_{d}}{M_{d}}\times 100$$

where, M_s_ is the mass of the swollen hydrogels and M_d_ is the mass of the dried hydrogels.

#### 2.3.5. Morphology of Hydrogels 

To investigate the surface and internal morphologies, hydrogels were frozen in liquefied nitrogen for 5 min, and the stored at −80 °C for 1 d before being lyophilized for 3 d. The freeze-dried hydrogels were again immersed in liquid nitrogen and were cut vertically, and then their surfaces and cross-sections were observed by using a SEM.

#### 2.3.6. Drug Loading and Release Studies

To prepare drug-loaded hydrogels, 1 mg/mL curcumin solution was incorporated with solid HA-Nb to obtain 2% solution of the polymer, followed by stirring until a homogenous mixture was obtained. Next, 200 μL of the curcumin-HA-Nb solution was mixed with 35 μL of a PEG-DTz solution (dissolved in DI water at various compositions ranging from 10 mg/mL, 20 mg/mL, and 40 mg/mL) in an Eppendorf tube. Then a vortex was used to gently mix the suspension for 15 s before being incubated in dark for 12 h at RT. Next, the curcumin-loaded hydrogels were rinsed with 3 mL of 20% solution of ethyl alcohol in DI water to remove un-loaded or surface-adhering curcumin. The absorptions of rinsing solutions were noted using a UV-Vis spectrophotometer (430 nm wavelength) to measure the drug loading efficiencies (DLE%) via a standard curve of curcumin. Curcumin loading efficiency was measured by using the following equation:

(2) $$\mathrm{DLE}\;\left(\%\right)=\frac{\mathrm{quantity}\;\mathrm{of}\;\mathrm{curcumin}\;\mathrm{in}\;\mathrm{feed}\;-\;\mathrm{quantity}\;\mathrm{of}\;\mathrm{curcumin}\;\mathrm{in}\;\mathrm{supernatant}}{\mathrm{quantity}\;\mathrm{of}\;\mathrm{curcumin}\;\mathrm{in}\;\mathrm{feed}}\times 100$$

The in vitro drug release evaluations of curcumin-loaded hydrogels were carried out by immersing them in a release medium (35 mL) containing 20% ethanol and 80% DI water. The samples were placed in a shaking incubator at 37 °C under moderate shaking (50 rpm). At a specific time-interval, 2 mL solution from the release medium was taken and analyzed by a UV-Vis spectrophotometer to measure the amount of the released curcumin. After measuring the absorbance at 430 nm wavelength, the withdrawn sample was placed back into the respective vial to maintain a constant volume.

#### 2.3.7. In Vitro Cytocompatibility of Precursors and Hydrogels

Cell compatibilities of HA-Nb, PEG-DTz, and the resulting hydrogels were studied in human embryonic kidney cells (HEK-293). Briefly, cells were seeded in a 48-well plate using Dulbecco’s Modified Eagle Medium (DMEM) along with 10% fetal bovine serum (FBS) and 1% Antibiotic-Antimycotic solution (Gibco™) at cell seeding densities of 10,000 cells/well, 37 °C, and 5% CO_2_ for 24 h. Next, the cell-medium was removed and exchanged with a fresh cell-medium containing different amounts of HA-Nb (0, 100, 500, 1000, 1500 and 2000 μg/mL) or PEG-DTz (0, 10, 20, 30, 40, and 50 μg/mL). After incubation for 24 h, cells were rinsed with 2 × 100 μL PBS, and were treated with 10 μL of a WST assay solution (EZ-cytox, Seoul, Korea). The absorbance of the resulting purple solution (formazan) was calculated using a microplate reader (wavelength 450 nm). Similarly, the cell compatibilities of the blank hydrogels were evaluated using hydrogel extracts.

## 3. Results and Discussion

### 3.1. Synthesis of PEG-DTz

**Figure 1:**
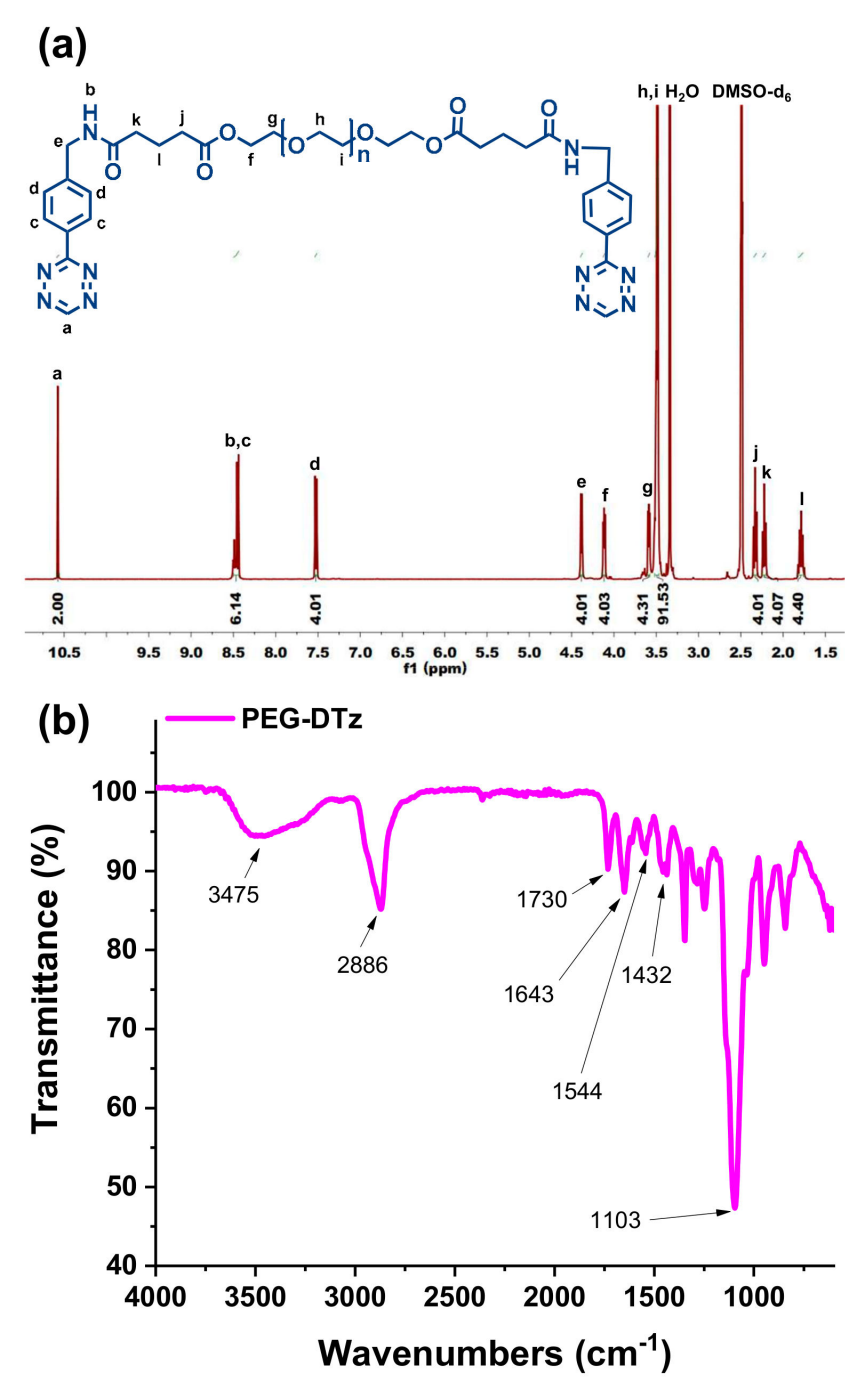
Characterization of the PEG-DTz cross-linker. (**a**) ^1^H NMR spectrum and (**b**) FTIR spectrum.

PEG-DTz was synthesized as shown in Figure 1. To develop the IEDDA click-able cross-linker, the carboxylic acid group of Tz-COOH and the hydroxy group of OH-PEG-OH were reacted via the carbodiimide coupling reaction forming ester linkages. The chemical structure of the PEG-DTz was analyzed by ^1^H NMR as shown in Figure 1 and further confirmed by ^13^C NMR spectroscopy (Supplementary Materials Figure S3).

The ^1^H NMR peak at 4.13 ppm indicates the protons next to the ester linkage, representing successful occurrence of the reaction. Whereas, repeating ethylene protons of PEG can be identified by the peaks at 3.51 ppm. The structure of the PEG-DTz was further validated by FTIR, demonstrating the C-H stretching of aromatic rings at 3475 cm^−1^ and the C-H stretching of PEG at 2886 cm^−1^. The C=O stretching of carbonyl groups was observed at 1730 cm^−1^. Whereas C=N, C-N, and C=C stretching vibrations of tetrazine molecules were noted at 1643 cm^−1^, 1544 cm^−1^, and 1432 cm^−1^, respectively. The stretching at 1103 cm^−1^ could be allocated to the C-O group of the PEG, demonstrating the successful synthesis of PEG-DTz.

### 3.2. Synthesis of HA-Nb

**Figure 2:**
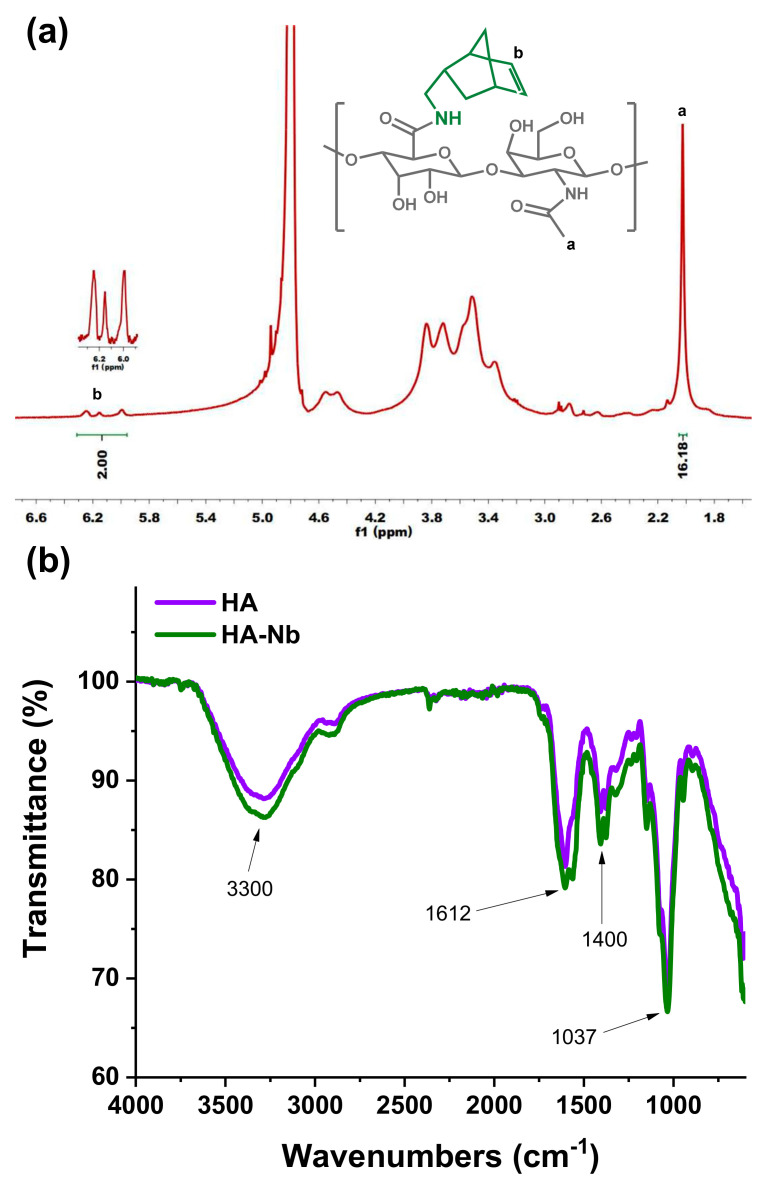
Characterization of HA-Nb. (**a**) ^1^H NMR spectrum of HA-Nb and (**b**) FTIR spectrum of HA and HA-Nb.

HA was equipped with Nb groups to prepare hydrogels by IEDDA click reaction with the Tz functional groups of PEG-DTZ. Specifically, HA underwent a functionalization reaction through the carbodiimide coupling reaction with Nb-NH2 to produce HA-Nb. The synthesized HA-Nb was assessed by a ^1^H NMR spectrometer (Figure 2a), showing a characteristic chemical shift of alkene protons of Nb moieties at 6.0–6.20 ppm.

The degree of substitution (DS) of Nb was calculated by equating the integrals of Nb protons with those of the methyl protons of HA, and was noted to be 18.55%. The successful synthesis of HA-Nb was further validated by FTIR (Figure 2b), displaying the OH stretching at 3300 cm^−1^, and the C=O stretching of asymmetric rings at 1612 cm^−1^, and the C=O stretching of symmetric rings at 1400 cm^−1^, and the C-O stretching at 1037 cm^−1^, confirming the successful functionalization of HA with Nb groups.

### 3.3. Preparation and Characterization of Hydrogels

**Figure 3:**
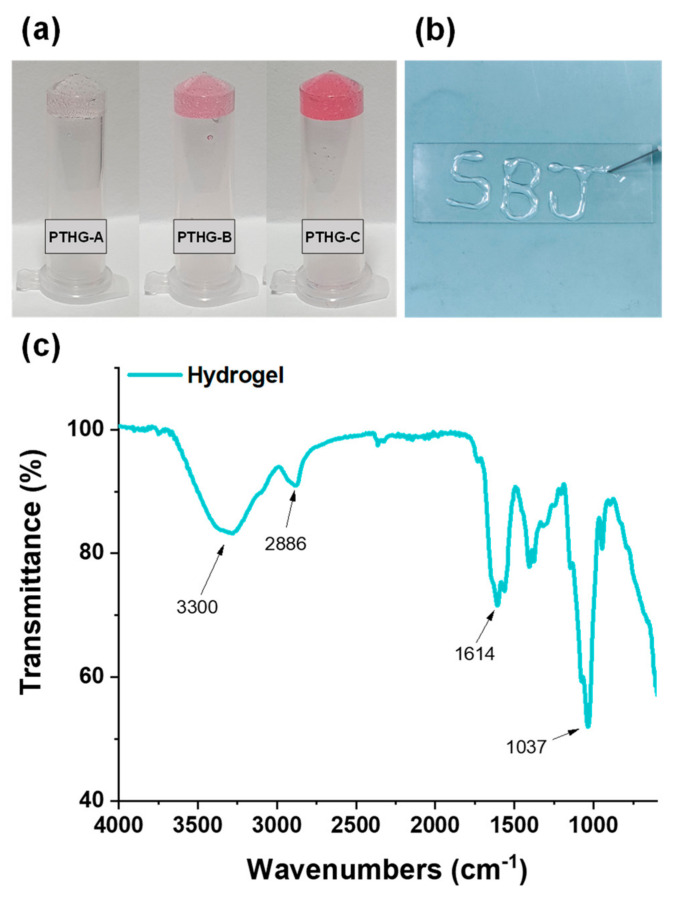
Prepared hydrogels. (**a**) Photographs of hydrogels (from left to right, PTHG-A, PTHG-B, PTHG-C, respectively), (**b**) Photograph showing injectability of hydrogel (PTHG-B), and (**c**) FTIR spectrum of PTHG-B.

Three types of hydrogels were developed coded as PTHG-A, PTHG-B, and PTHG-C, by varying the Nb:Tz mol. ratio (10:2.5, 10:5, and 10:10, respectively) in the formulations. The IEDDA click reaction occurred spontaneously and transformed the polymer sol into gel, producing stable hydrogels, as shown by the photographs (Figure 3a) acquired at the end of the cross-linking reaction. The IEDDA cycloaddition reaction is commonly employed for proficient and selective bio-conjugation [26]. Among the reagents employed in the IEDDA reaction, Tz and Nb react powerfully and promptly under mild and catalyst-free environment to produce small quantities of nitrogen but no cytotoxic byproduct. The reaction is also known for its rapid kinetics and good quantitative yield, and can take place even in water or biological fluids. The IEDDA reaction cross-linked HA, while the liberated nitrogen gas generated microbubbles and porosity in the gel networks. The IEDDA reaction was fast enough to produce injectable hydrogels (Figure 3b). The hydrogels were characterized by a FTIR (Figure 3c), showing all the characteristics peaks of the HA and PEG-DTz, confirming the formation of hydrogels by IEDDA click reaction.

#### 3.3.1. Gelation Times and Mechanical Properties of Hydrogels

**Figure 4:**
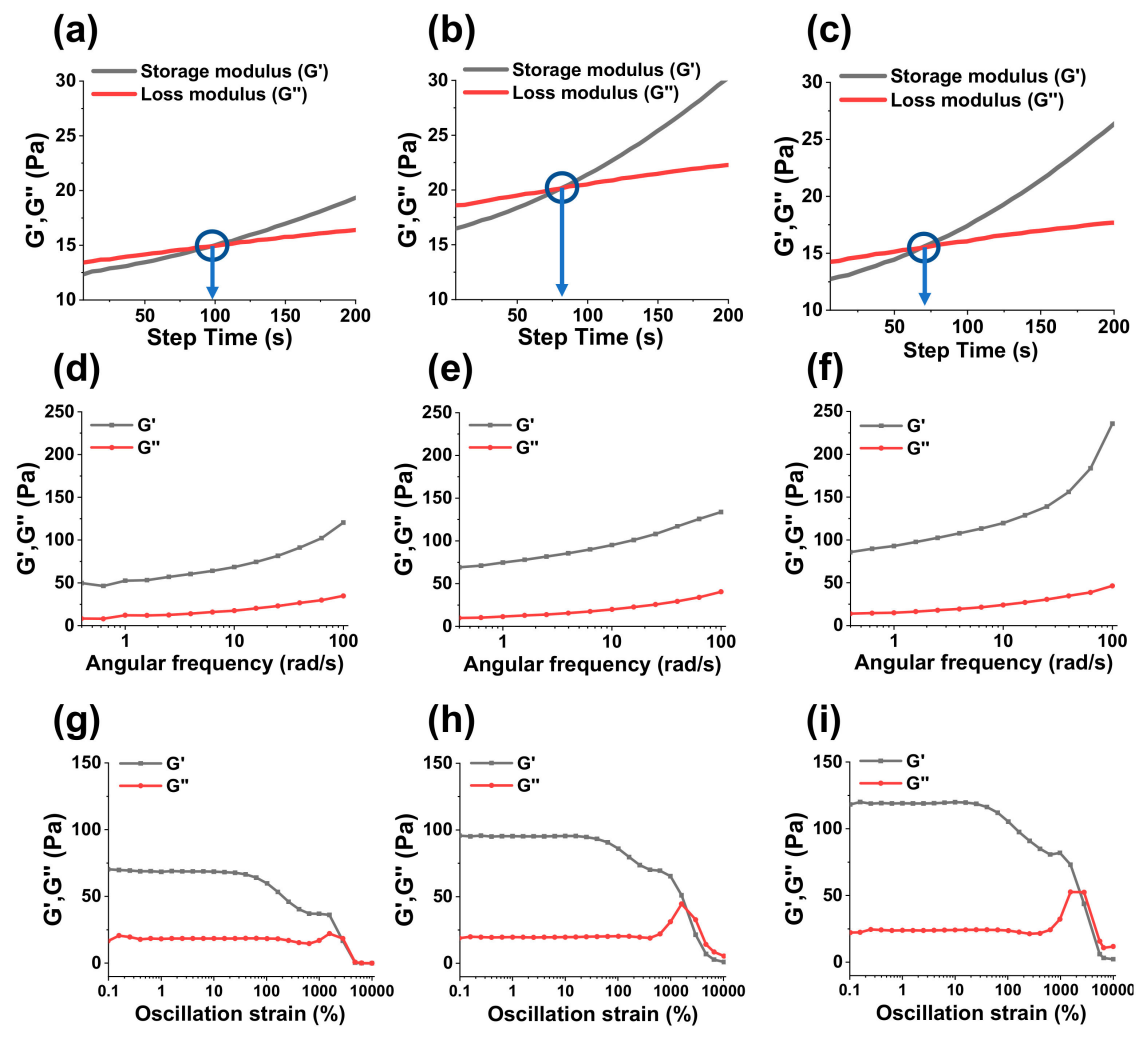
Rheological properties of PTHG-A, PTHG-B, and PTHG-C hydrogels. (**a**–**c**) storage modulus (G’) and loss modulus (G”) as a function of gelation time, (**d**–**f**) angular frequency sweep tests, and (**g**–**i**) amplitude sweep tests.

The sol-gel conversion time and viscoelastic behavior of hydrogels were evaluated by using a rheometer. The G“ and G” of hydrogels were analyzed as a function of step time and angular frequency sweep, and amplitude sweep test. The step-time experiment showed that all three types of hydrogels transformed rapidly from sol to gel as shown by the intersection of G“ and G”. Specifically, PTHG-A, PTHG-B and PTHG-C formulations just took about 99, 81, and 68 s to change into gels (as shown in Figure 4a–c, respectively), demonstrating that the gelation time of the hydrogel decreases as the content of the cross-linker increases. The angular frequency sweep test demonstrated the G“ values of about 120, 134, and 236 Pa for PTHG-A, PTHG-B, PTHG-C, as shown in Figure 4d–f, respectively. These viscoelastic findings are consistent with the previously reported data, which confirmed that the content of the cross-linker could increase the cross-linking density of hydrogels and therefore, the mechanical strength of hydrogels increased [27]. The amplitude-sweep tests showed that PTHG-A, PTHG-B, and PTHG-C have a gradual shear thinning of the hydrogels as shown by a decrease in their G“ values (Figure 4g–i, respectively) after increasing the strain up to 1000%. This result indicated that the formulated hydrogels possessed shear thinning behaviors and could be easily extruded through a needle for injectable drug delivery or 3D printing applications.

#### 3.3.2. Swelling Behavior of Hydrogels

**Figure 5:**
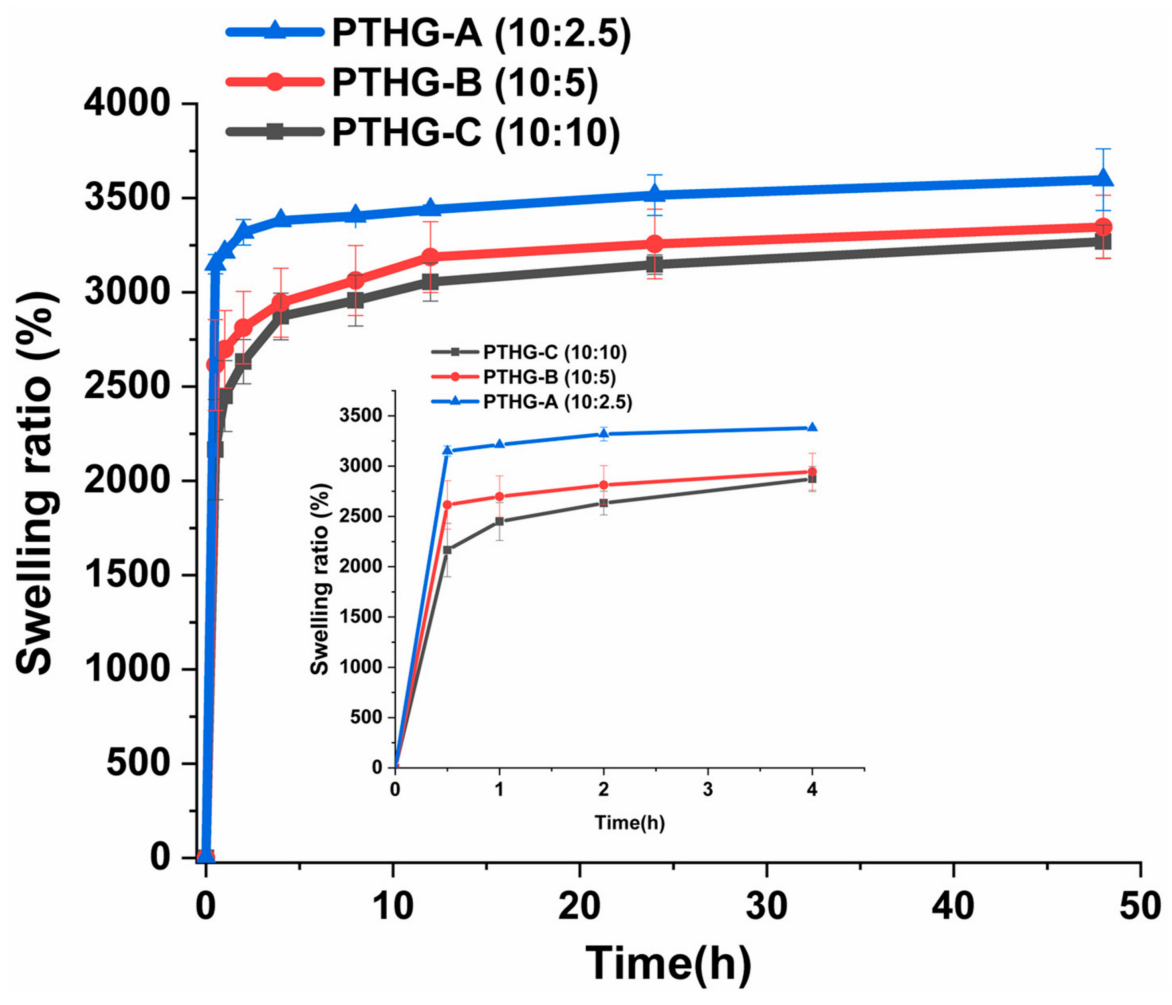
Swelling properties of hydrogels in PBS (pH 7.4). Inset showing swelling of hydrogels during initial 4 h. Error bars showing standard deviation (n = 3).

The prepared hydrogels were lyophilized to study their swelling properties. The hydrogels were submerged in 3 mL of PBS (pH = 7.4) and the weights of the swollen hydrogel were measured at predetermined time to calculate their swelling rates. All the formulated hydrogels swelled rapidly just in 30 min after their immersion in PBS, as shown in Figure 5. This rapid swelling could be attributed to the presence of hydrophilic PEG chains in the cross-linked bridges of the hydrogels. The highly porous architectures could also be responsible for the fast and instant swelling of the hydrogels. The rapid swelling phase was followed by a consistent and sequential increase in swelling ratios to reach a swelling equilibrium after 48 h. The maximum swelling ratios of PTHG-A, PTHG-B, and PTHG-C hydrogels were about 3596, 3346, and 3268% after 48 h, respectively, revealing that the higher degree of cross-linking decreased the swelling of the hydrogels. This trend in swelling of the hydrogels was consistent with the previous reported data [28]. The swelling behavior of as prepared hydrogels (without lyophilization) was also checked to know whether hydrogels would swell significantly or not after injection. Interestingly, the volume of hydrogels did not expand significantly as compared to the lyophilized hydrogels, as shown in Supplementary Materials Figure S4.

#### 3.3.3. Morphology of Hydrogels

**Figure 6:**
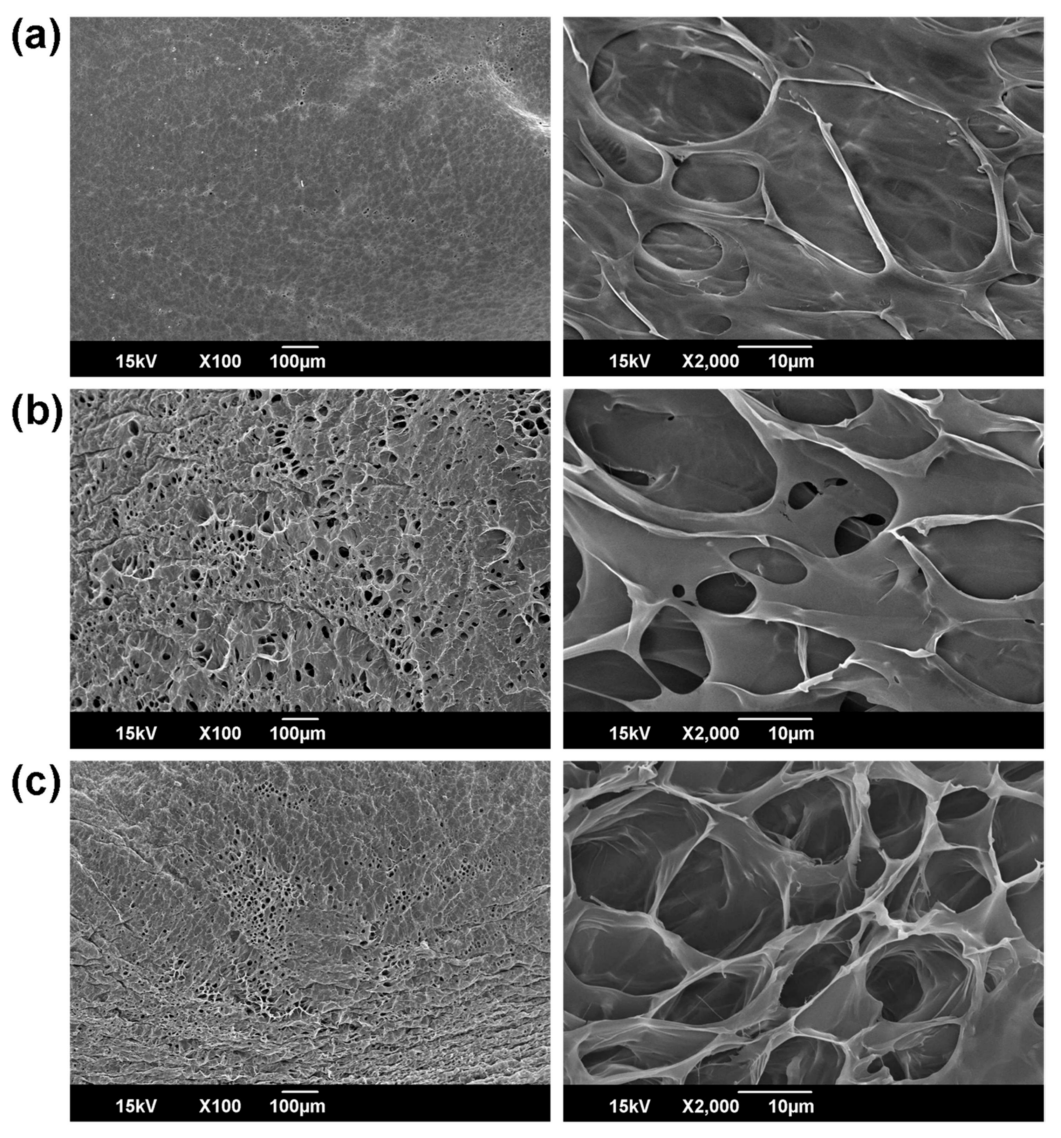
Scanning electron microscopic (SEM) images of surface (left side images) and the cross-section (right side images) of hydrogels. (**a**) PTHG-A, (**b**) PTHG-B, and (**c**) PTHG-C.

The surfaces and internal structures of the hydrogels were observed by means of a SEM. As can be seen in Figure 6, all the hydrogels displayed highly porous morphologies with pore presented on both the surfaces and in the cross-sectional areas of the hydrogels. These surface-pores could be credited to the N_2_ gas liberated during the IEDDA reaction, whereas the internal pores could be due to the N_2_ gas as well as cross-linked networks. Interestingly, PTHG-A with a lower cross-linker content exhibited a looser structure and larger sized pores, whereas PTHG-C with a higher cross-linker content showed a denser structure and smaller sized pores, again attributed to the difference in their crosslinking densities. Since lyophilization could also produce pores, non-lyophilized wet hydrogels were observed by a digital camera in order to confirm the pores were initially generated by the nitrogen gas produced during IEDDA reaction. As expected, Supplementary Materials Figure S5 shows microbubbles formed in the as prepared hydrogels.

### 3.4. Drug Loading and Release Studies

**Figure 7:**
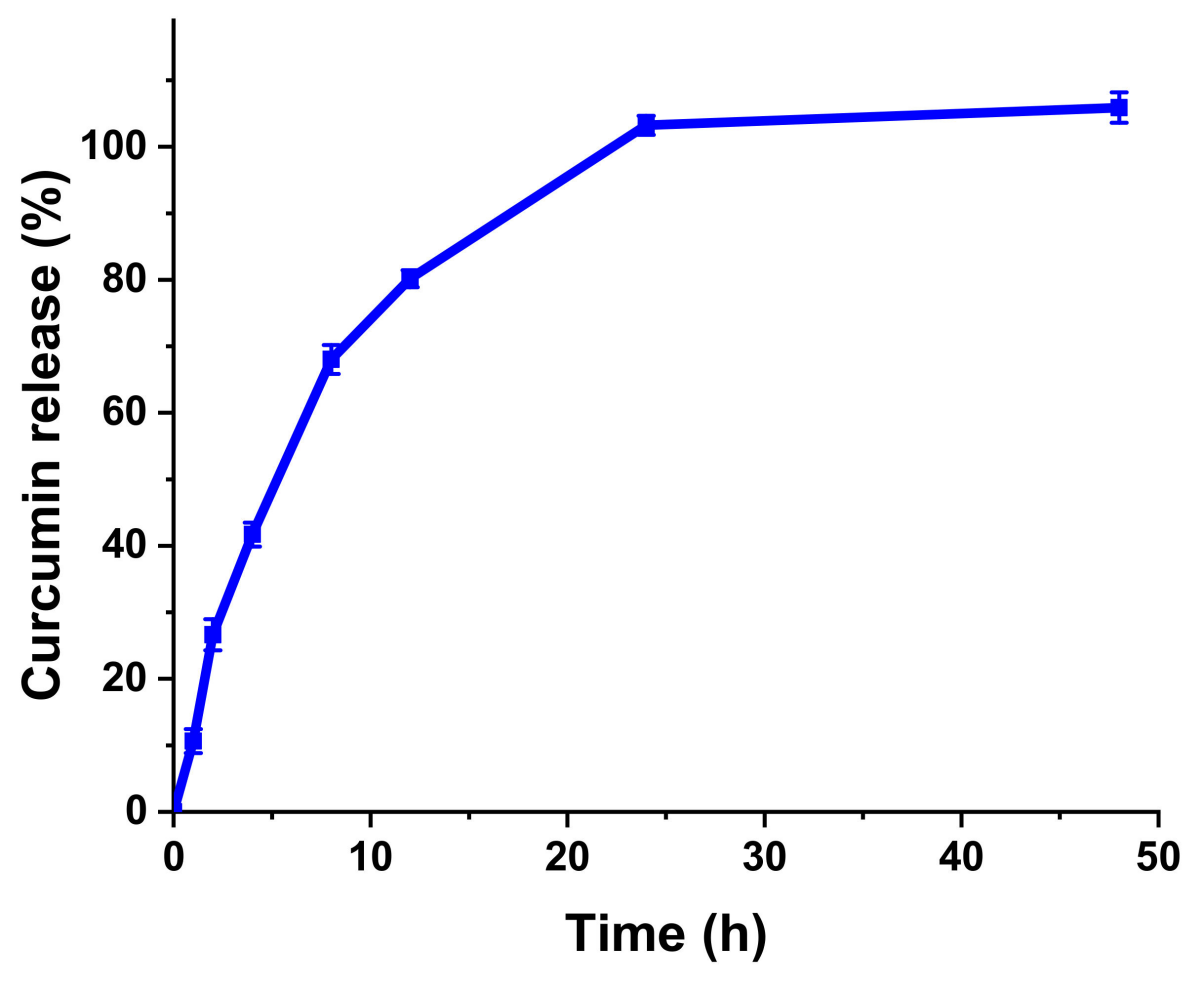
In vitro curcumin release behavior of PTHG-B in PBS containing 20% ethanol. Error bars showing standard deviation (n = 3).

The PTHG-B (Nb:Tz = 10:5) hydrogel with medium swelling ratio and mechanical properties was selected to assess the drug loading efficiency and drug release behavior. As HA-based hydrogels fabricated with PEG-DTz showed a rapid swelling and highly porous structures, it was expected to exhibit fast drug release. The drug loading (DLE%) and release studies were performed using 1 mg/mL curcumin. Interestingly, the DLE% of the hydrogels was remarkably high (about 99%), which is probably due to the highly porous structure of the hydrogels, which facilitated the encapsulation of curcumin within cavities of the hydrogels. Since curcumin is a therapeutic agent with quite low bioavailability and high hydrophobicity, therefore, drug release experiments were performed in PBS containing 20% ethanol. The PTHG-B hydrogels release about 10.6% curcumin after 1 h and 26.6% after just 2 h (Figure 7). This fast drug release could be due to the rapid swelling and highly porous morphologies of the hydrogels. The fast release phase was followed by a sequential drug release phase and the PTHG-B hydrogel released most of encapsulated drugs (100% drug release) after 48 h. This type of drug release without employing any complicated systems could be beneficial for bioorthogonal and injectable drug delivery systems. The result was similar to the previously reported IEDDA cross-linked hydrogels based on HA-Tz and PEG-Nb as hydrogel precursors for in situ encapsulation of Fab1 proteins, which demonstrated the complete release of FAB1 from the hydrogel matrix over a period of several weeks [29].

### 3.5. In Vitro Cytotoxicity of Precursors and Blank Hydrogels

**Figure 8:**
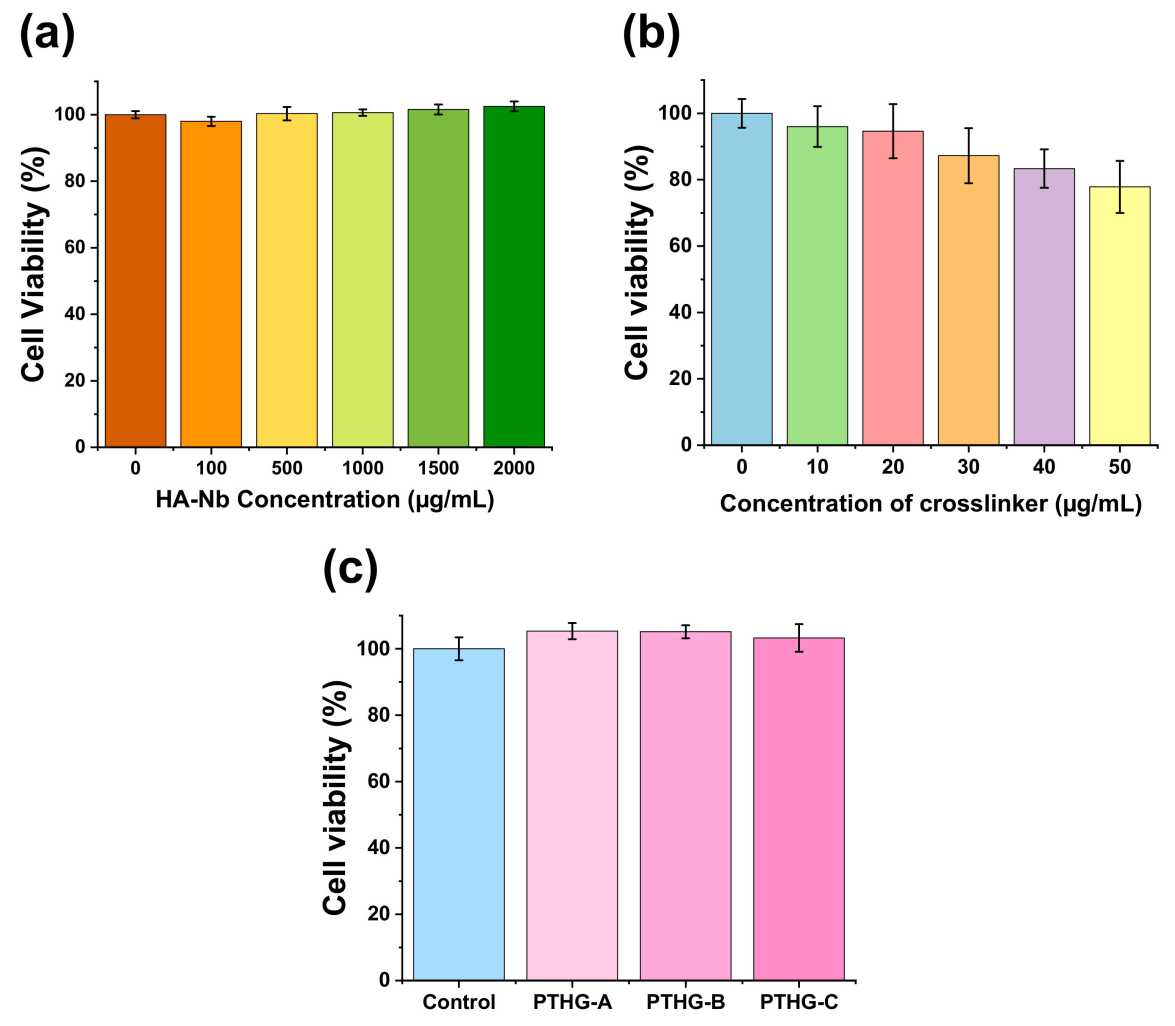
In vitro cytocompatibility of HEK-293 cells after treatment with (**a**) HA-Nb, (**b**) PEG-DTz, and (**c**) Hydrogels (PTHG-A, PTHG-B, and PTHG-C). Error bars showing standard deviation (n = 4).

The cross-linker, HA-Nb, and the resulting hydrogels should not be cytotoxic for biomedical applications. The cytotoxicity of PEG-DTz, HA-Nb, and blank hydrogels were evaluated to examine their cytocompatibility. HEK-293 cells were selected as a normal cell model and cytotoxicities were evaluated by using WST assays. For cytotoxicity evaluation, HE-293 cells were incubated with different concentrations of PEG-DTz, HA-Nb, and blank hydrogels (PTHG-A, PTHG-B, PTHG-C) for 24 h. Figure 8 represents the results of cytotoxicity evaluation. HA-Nb was highly biocompatible and did not show any cytotoxicity in HEK-293 cells up to concentrations of 2000 μg/mL, as shown in Figure 8a. The PEG-DTz cross-linker was also cytocompatible (Figure 8b) up to 50 μg/mL concentrations (>80% cell viability). Furthermore, the cytotoxicity evaluation results of blank hydrogels showed that they were also highly biocompatible as no cytotoxicity was observed after treating HEK-293 cells with PTHG-A, PTHG-B, and PTHG-C hydrogels. These results indicate that the porous hydrogels prepared by IEDDA click cross-linking reaction were highly biocompatible, as no by-product (except non-toxic nitrogen) is generated during this reaction. Moreover, IEDDA reaction does not require any catalyst, can take place at physiological conditions, and in biological fluids. Therefore, these biocompatible hydrogels with rapid swelling behavior and highly porous morphologies could be useful candidates for injectable and controlled drug release applications.

## 4. Conclusions

The IEDDA cross-linked hydrogels having injectable properties and fast absorbent behavior, were developed. The porosity, gelation times, and mechanical strength of the hydrogels were programmed by changing the mol. ratio of the cross-linker. Owing to the highly hydrophilic nature of the cross-linker and the porous micro-architectures, the hydrogels swelled instantly. The hydrogel formulation (PTHG-B) showed great encapsulation efficiency (~99%) of curcumin, which was released rapidly during the initial phase, and then a sustained drug release from the hydrogel was noted. The resulting hydrogels were highly biocompatible with the tested cell line (HEK-293), showing a highly bioorthogonal nature of the IEDDA-cross-linked hydrogels. These fast swelling and highly porous hydrogels could be useful candidates for injectable drug delivery applications.

## Figures and Tables

**Table 1:** Composition of hydrogels and their gelation times.

Sample	Nb:Tz Ratio (Moles)	Gelation Time (s) ^1^
PTHG-A	10: 2.5	99
PTHG-B	10: 5	81
PTHG-C	10: 10	68

^1^ gelation time was calculated from the intersection of G’ and G” by using a rheometer.

## Data Availability

The data presented in this study are available upon request from the corresponding author.

## Supplementary Materials

The following supporting information can be downloaded at: https://www.mdpi.com/article/10.3390/ma15207128/s1, Figure S1: Characterization of (4-(cyano) benzylamino)-5-oxopentanoic acid. (a) 1H NMR spectrum and (b) ^13^C NMR spectrum, Figure S2: Characterization of tetrazine(benzylamino)-5-oxopentanoic acid (Tetrazine-COOH). (a) ^1^H NMR spectrum and (b) ^13^C NMR spectrum, Figure S3: ^13^C NMR of PEG-DTz, Figure S4: Swelling properties of non-lyophilized hydrogels in PBS (pH 7.4), Figure S5: Photographs of hydrogels with microbubbles (from left to right, PTHG-A, PTHG-B, and PTHG-C).
