# Supplementary material for: Fast Absorbent and Highly Bioorthogonal Hydrogels Developed by IEDDA Click Reaction for Drug Delivery Application
Source: Materials (Basel). 2022 Oct 13;15(20):7128. doi: 10.3390/ma15207128 (PMC9608709; doi:10.3390/ma15207128)
Supplement: Supplementary file 1 [file materials-15-07128-s001.zip › materials-1947195-supplementary.pdf]

Supporting Information

Fast Absorbent and Highly Bioorthogonal Hydrogels Developed by IEDDA Click Reaction for Drug Delivery Application

Soo-Bin Joo[1], *Muhammad Gulfram*[1], *Sung-Han Jo*[2], *Yi-Jun Jo*[1], *Trung Thang Vu*[1], *Sang-Hyug Park*[2], *Yeong-Soon Gal*[3,*], *Kwon Taek Lim*[1,*]

[1] Department of Industry 4.0 Convergence Engineering, Pukyong National University, Busan 48513, Korea

[2] Department of Biomedical Engineering, Pukyong National University, Busan 48513, Korea

[3] Department of Display Engineering, Pukyong National University, Busan 48513, Korea

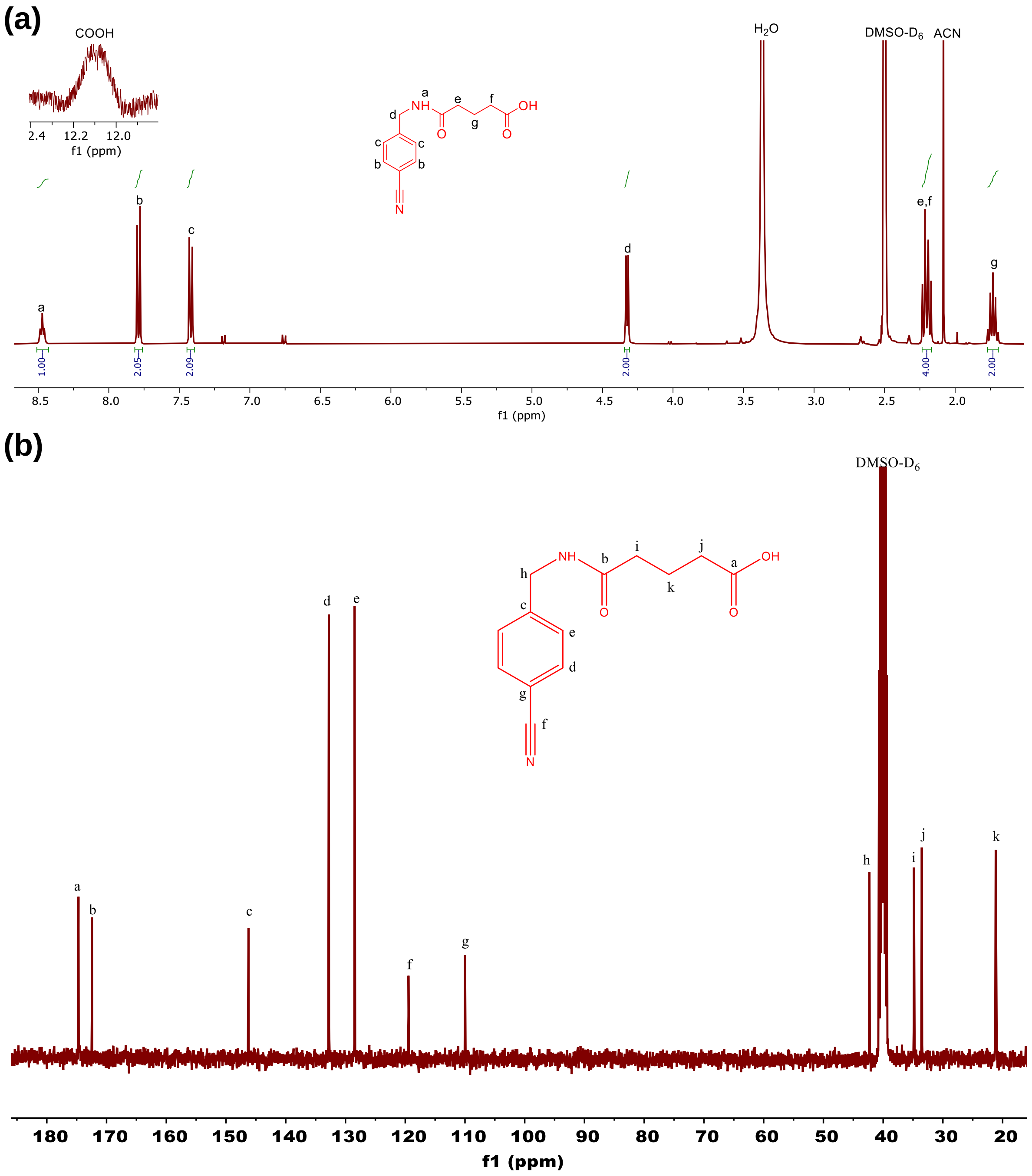

Figure S1. Characterization of (4-(cyano) benzylamino)-5-oxopentanoic acid. (a) ^{1}H NMR spectrum and (b) ^{13}C NMR spectrum.

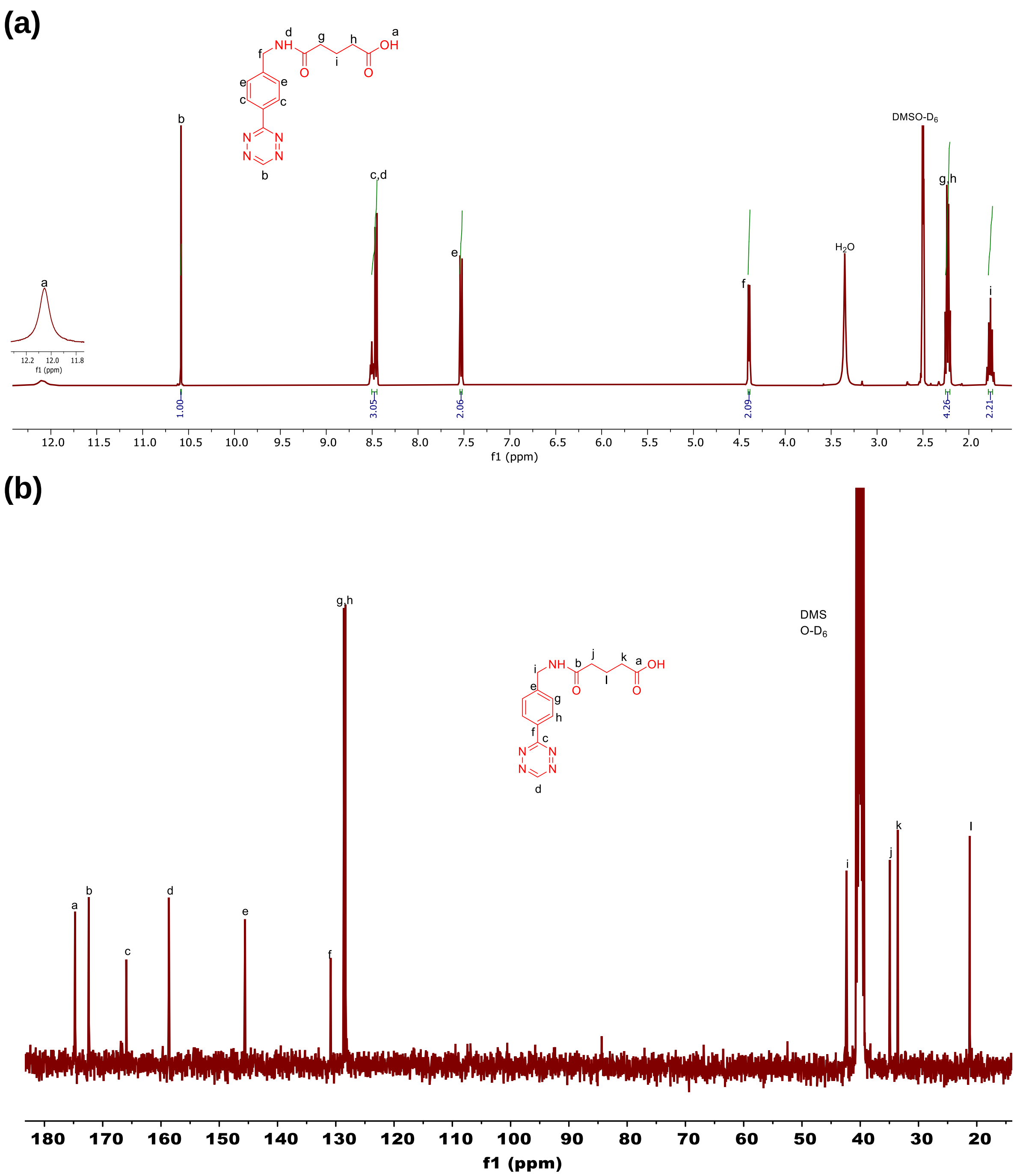

Figure S2. Characterization of tetrazine(benzylamino)-5-oxopentanoic acid (Tetrazine-COOH). (a) ^{1}H NMR spectrum and (b) ^{13}C NMR spectrum.

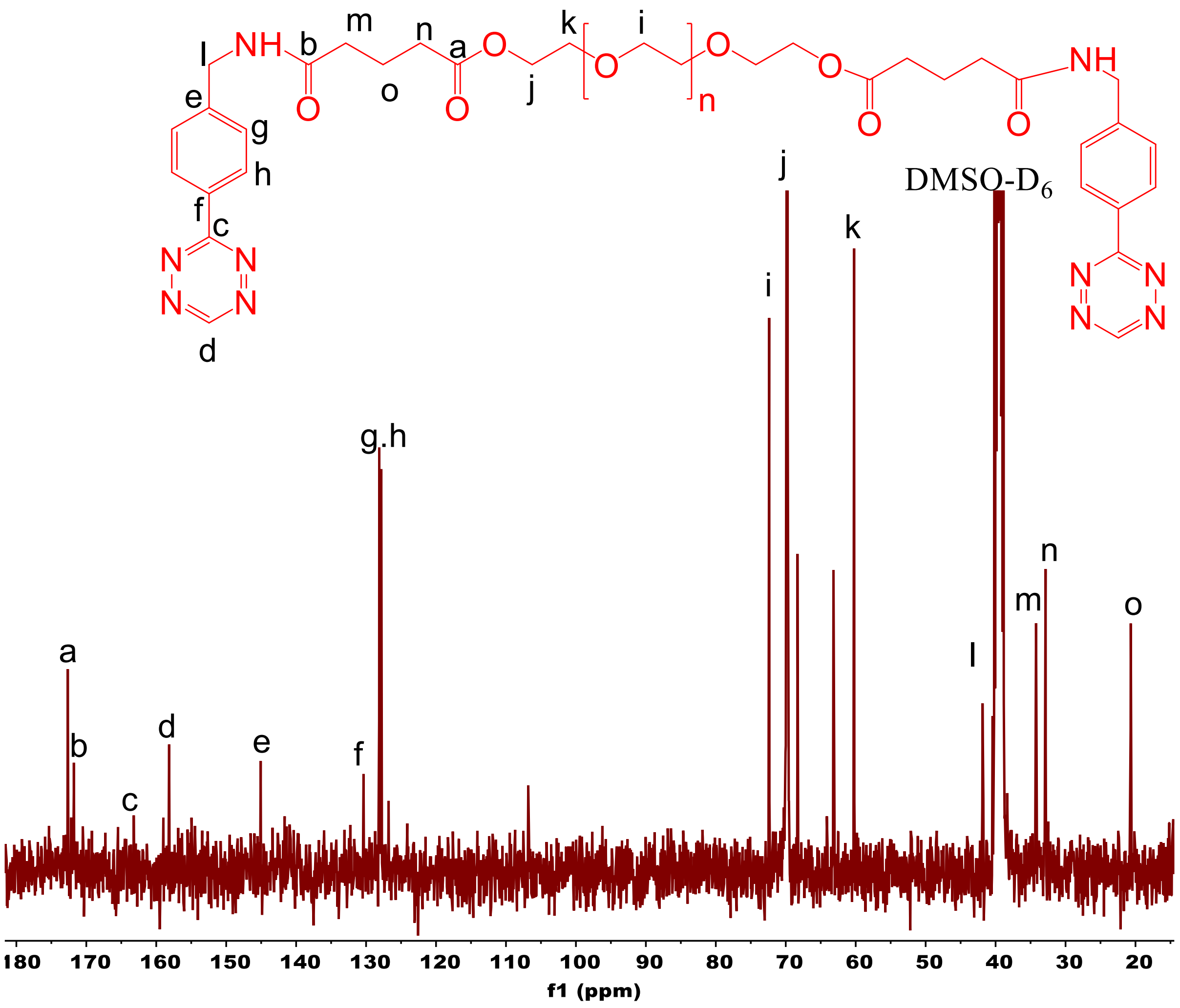

Figure S3. ^{13}C NMR of PEG-DTz.

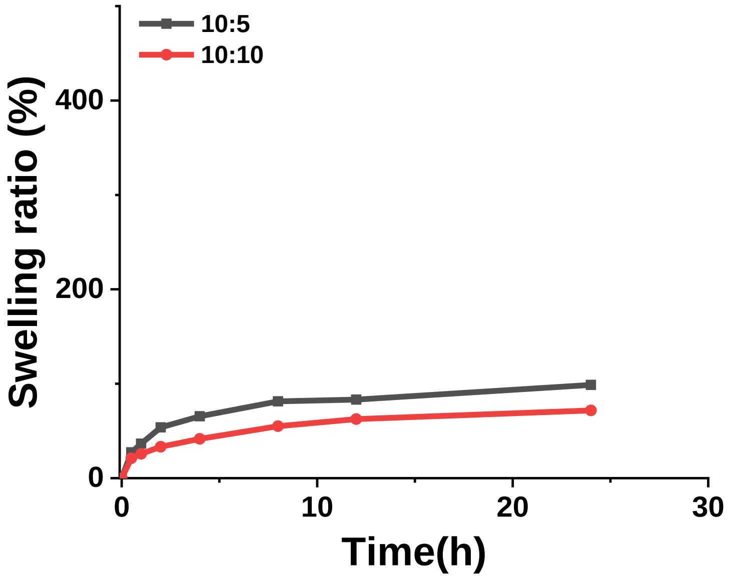

Figure S4. Swelling properties of non-lyophilized hydrogels in PBS (pH 7.4).

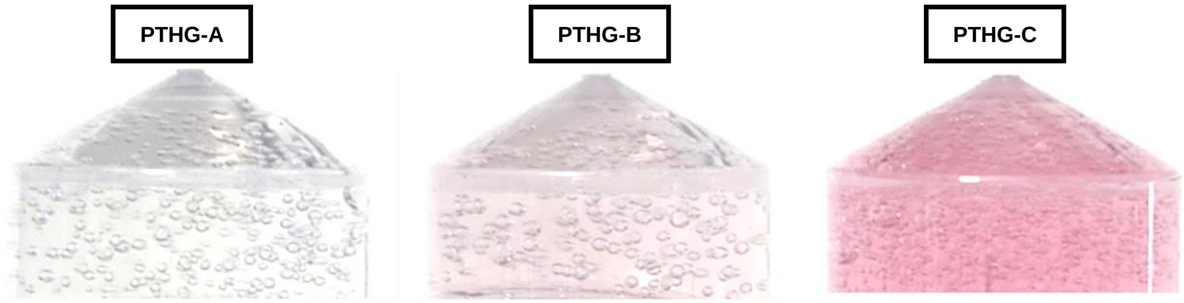

Figure S5. Photographs of hydrogels with microbubbles (from left to right, PTHG-A, PTHG-B, and PTHG-C).
